# Estimation of yield and nitrogen use efficiencies in hybrid maize varieties through site specific nitrogen management based on leaf color chart (LCC)

**DOI:** 10.1038/s41598-025-89393-3

**Published:** 2025-02-08

**Authors:** Suhail Fayaz, Raihana Habib Kanth, Tauseef A. Bhat, Eajaz Ahmad Dar, Zahoor Ahmad Shah, Moneesa Bashir, Aijaz Nazir, Bilkees Jamsheed, Mohd. Salim Mir, Zahoor A. Dar, Shailja Sharma, Aabid Hussain Lone, Dawood Yousuf, Nadhir Al-Ansari, Mohamed A. Mattar, Ali Salem

**Affiliations:** 1 Division of Agronomy, Faculty of Agriculture, Sher-e-Kashmir University of Agricultural Sciences and Technology of Kashmir, Wadura, Sopore, 193201 India; 2 Dean Faculty of Agriculture, Sher-e-Kashmir University of Agricultural Sciences and Technology of Kashmir, Wadura, Sopore, 193201 India; 3 Sher-e-Kashmir University of Agricultural Sciences and Technology of Kashmir, Srinagar, 190006 Jammu and Kashmir India; 4 Department of Agricultural and Biological Engineering, University of Florida, Frazier Rogers Hall, PO Box 110570, Gainesville, FL 32611-0570 USA; 5 Division of Agri. Extension, Faculty of Agriculture, Sher-e-Kashmir University of Agricultural Sciences and Technology of Kashmir, Srinagar, 190025 Jammu and Kashmir India; 6 Dryland Agriculture Research station, Sher-e-Kashmir University of Agricultural Sciences and Technology of Kashmir, Shalimar, 190025 Srinagar India; 7 School of Agriculture, Division of agronomy, Lovely Professional University, Ludhiana, 144411 India; 8 Mountain Research Centre for Field Crops- Khudwani, Sher-e-Kashmir University of Agricultural Sciences and Technology of Kashmir, Shalimar Srinagar, Jammu and Kashmir India; 9 ARSSSS, Sher-e-Kashmir University of Agricultural Sciences and Technology of Kashmir, Shalimar Srinagar, Dussu, Pampore, India; 10 Department of Civil, Environmental, and Natural Resources Engineering, Lulea University of Technology, Lulea, 97187 Sweden; 11 Department of Agricultural Engineering, College of Food and Agriculture Sciences, King Saud University, P.O. Box 2460, Riyadh, 11451 Saudi Arabia; 12 Structural Diagnostics and Analysis Research Group, Faculty of Engineering and Information Technology, University of Pécs, Pécs 7622, Hungary; 13 Civil Engineering Department, Faculty of Engineering, Minia University, Minia 61111, Egypt

**Keywords:** Economics, Hybrids, LCC, Maize, N Management, Nitrogen use efficiency, Yield, Agroecology, Agroecology

## Abstract

Exorbitant praxis of nitrogen pioneered and opened up the usage of time-specific and need-based nitrogen management. The leaf color chart (LCC), being one of the handy tool, is put to use for the estimation of the indirect leaf N, and also heightens the competence of crop N administration. Integrating leaf color chart assessments into maize cultivation practices can provide a practical and cost-effective approach for tailoring nitrogen applications, leading to improved resource-use efficiency and sustainable maize production. To perceive the specific leaf color chart (LCC) value for precision maneuvering of nitrogen in different maize hybrids, a field experiment was carried out at the research farm of Division of Agronomy, FoA, Wadura, Sopore, SKUAST-Kashmir, in the years 2019 and 2020 (*Kharif* seasons). Split Plot Design (SPD) employing three maize varieties (Shalimar Maize Hybrid-2 (SMH-2), Kanchan-517 and Vivek-45) in main plots and precision management of nitrogen (T_1_: Control, T_2_: Recommended Nitrogen (120 kg N ha^–1^), T_3_: 25% N as basal LCC @20 (≤ 3) kg N ha^−1^, T_4_: 25% N as basal LCC @30 (≤ 3) kg N ha^−1^, T_5_: 25% N as basal LCC @20 (≤ 4) kg N ha^−1^, T_6_: 25% N as basal LCC @30 (≤ 4) kg N ha^−1^, T_7_: 25% N as basal LCC @20 (≤ 5) kg N ha^−1^ and T_8_: 25% N as basal LCC @30 (≤ 5) kg N ha^−1^) in sub-plots was systemized. The pooled means indicated that SMH-2 chalked up utmost values in growth (plant height 189.4 cm) and periodic leaf area index and yield parameters with a grain yield of 6.1 t ha^–1^, straw yield of 10.6 t ha^–1^, respectively. The LCC value of @ 30 (≤ 5) kg N ha^–1^ set down statistically highest and significant grain (6.0 t ha^–1^) and straw yield (10.8 t ha^–1^) pooled over the years amidst other nitrogen management treatments, respectively. LCC @ 20 (≤ 5) kg N ha^–1^ turned up to have highest apparent N recovery (REN) followed by LCC @ 30 (≤ 5) kg N ha^-1^ whereas LCC @ 20 (≤ 4) kg N ha^–1^ which was at par with LCC @ 20 (≤ 5) kg N ha^-1^ recorded maximum agronomic efficiency (AE). LCC @ 20 (≤ 3) kg N ha^–1^ showed the highest physiological efficiency (PE) and partial factor productivity (Pfp). Furthermore, different rice genotypes manifested significant effects vis-à-vis Pfp and REN, which were maximum in SMH-2 contrasted with Kanchan-517 and Vivek-45. In the interim, the economics of pooled data divulged that the maximum B: C ratio was observed in SMH-2 and LCC @ 30 (≤ 5) kg N ha^–1^. Therefore, site-specific nutrient management through LCC proved to be an effective strategy to maximize yield and nitrogen use efficiency in hybrid maize.

## Introduction

Maize is an important crop of the world for its great value in human and animal diets. It is cultivated on 197 million hectares globally with a production of 1137 million tons. The area and production of maize in India are 9.47 million hectares and 28.64 million tons^1^. Maize, the queen of cereals holding paramount importance in the union territory of Jammu and Kashmir, has a total acreage of 2.6 lakh hectares^2^. For years, its average production has approximated to twofold owing to the build-out of the area under high-yielding varieties. Proper fertilization of the crop has shown good outturn. Crop response to fertilizer changes across different locations subjected to environmental factors, soil fertility and genotypes. Maize crop in itself is exacting owing to exigency in soil nutrients particular to NPK. Nitrogen being indispensable to DNA composition and proteins backs cell division and cell reproduction^3^and thus bases maize beefing up in productivity. Nonetheless, imprudent application of nitrogen mayhem in humans is exclusive to ecosystem, thus necessitating nitrogen management in accordance with crop needs^4^, as system sustainability is corresponded by nutrient management methods. Nutrient management has also been proclaimed to be essential for self-sufficiency with regard to food grain production. Predicted average response of fertilizers in years and inadequate fertilization has contributed to nitrogen losses. Lopsided prophetic responses to contrasting N application levels in maize heighten the uncertainty in the optimum N rates, posing a danger in economics. Also, yield gaps in maize correlate with poor agro-management techniques. This uncertainty could be overcome by locating precise area and time-specific advice^5^. Proper dosage of fertilizers and the right time of application of fertilizers also add to the efficient usage of fertilizers^6^. Site-specific nutrient management (SSNM) is the one solution towards efficient nutrient management with respect to proper dosage and the right time of application of fertilizers at crucial growth phases to overcome yield gaps^7^. SSNM research has conveyed yield benefits above blanket recommendations on nutrient omission plots that have made clear understanding of the functionalities of specific nutrients in yield growth^8^. However, due to the loopholes in SSNM technology, farmers are reluctant to use it^8^. Prerequisite-based fertilizer N management employing a soil plant analysis development (SPAD) meter was a useful lead in the regulation of timely application of N^9^. Nonetheless, the high cost of SPAD makes it inaccessible to small and marginal farmers. The praxis of LCC has turned out to be cost-effective and unswerving in practice compared to the SPAD meter. It is effective in delivering the controlled nitrogen rates to rice and wheat crops^10^, as is the case with South Asian farming, and thus necessitates its usage in maize as well. The nutrient supplication to maize through site-specific nutrient management is based on the nutrient application at the right time with an accurate dose, which will enable the farmers to quantify fertilizer use dynamically for synchronizing the crop’s need for higher and optimum yields. Gabinete et al.^8^. also reported that site-specific nutrient management application recorded significantly higher yield and uptake of N, P, S and Zn as compared to conventional fertilization.

Existing research has primarily focused on conventional nitrogen management approaches, with limited attention given to the comparative effectiveness of leaf color charts (LCC). As the farmers generally prefer the application of higher quantities of nitrogen in maize, leading to low nitrogen recovery in crops, besides with the enhanced cost of production and environmental degradation, the leaf color chart (LCC) is one of the ideal tools for optimizing nitrogen use in maize based on the spectral reflectance of maize leaves. LCC helps in promoting real-time nitrogen application in maize by synchronizing nitrogen application and crop need. A research gap exists in systematically evaluating the accuracy and reliability of LCC in assessing nitrogen status, particularly in comparison to traditional methods like soil testing and plant tissue analysis^11^. While some studies have explored the utility of LCC in diagnosing nitrogen deficiency in maize, there is a research gap regarding its effectiveness under varying soil nutrient conditions and crop management practices. Investigating the adaptability of LCC to different agro-ecosystems would provide valuable insights into its practicality across diverse agricultural settings. The integration of LCC with precision agriculture technologies remains an unexplored area. A research gap exists in understanding how advanced tools such as drones, satellite imagery, or sensor-based systems can enhance the efficiency and scalability of LCC for real-time nitrogen management, offering potential solutions for precision farming^12^.

Leaf color chart (LCC) in maize gauges chlorophyll content in the leaf in a non-destructive and non-invasive way, thus furnishing leaf N status indirectly. It acts as a yardstick for the N level of the maize crop from the observations of the relative greenness of a leaf^13^. As a case with farmers, practically, it puts forward a notable look-in for arbitrating demand-based quantity and time of application of nitrogen for efficient usage in maize crop, as physical scrutiny of the intensity of leaf color in the case of standard LCC enlists the requirements of nitrogen in the plant with respect to time and assists in the management of scheduling top dressing of N to maize. Suhail et al.^14^. reported that application of nitrogen as per LCC 5 improved the growth and yield of hybrid maize. Also, Shivkumar and Gasavannepa^15^ found that precise application in maize through LCC threshold 5 was effective in enhancing the growth and yield of maize.

Union territory of Jammu and Kashmir has very high maize yield gaps owing to defective agro-management practices inclusive of unbalanced nutrient management as the prime factor. Spatial and temporal soil variability are also not taken into cognizance during the application of fertilizers. Currently, maize yields in valleys are underneath their potential yield as nitrogen management is improper, so devising techniques for need-based application of N in valleys will be a useful strategy for farmers in valleys. Hence, the aim of the research was to ascertain the critical values for nitrogen management in hybrid maize cultivars employing LCC.

## Materials and methods

The experimentation of the research work was done in the Agronomical Farm of the Agronomy Division, Faculty of Agriculture, Wadura, SKUAST-K, India, during the *Kharif* seasons in the years 2019 and 2020 to analyze the impact of the management of nitrogen through the leaf color chart (LCC) on different maize hybrids performance.

### Site of the experiment

The experiment was executed at the location having 1590 masl altitude amidst the latitude and longitude of 34°210 N and 74°230 E, respectively. Soil was having the texture of silty clay loam. The soil analysis revealed that the soil contained the available nitrogen, phosphorus and potassium as 320.5, 19.75 and 170.2 kg ha^–1^, respectively; pH was neutral with medium organic carbon (0.66%) (Table 1).

**Table 1:** Physico-chemical characteristics of the maize field.

Particulars	Value	Procedure employed
Physical analysis		
Sand (%	20.0	International pipette method^16^
Silt (%)	50.0	
Clay (%)	30.0	
Texture: Silt clay loam		
Bulk Density (Mgm^−3^)	1.3	Core sampler method^17,18^
Physico-chemical examination		
pH	7.1	Backman’s glass electrode pH meter^19^
Electrical conductivity (dsm^−1^) at 25 ^0^C	0.2	Conductivity meter^19^
Organic carbon (%)	0.6	Wet digestion method (rapid titration Method^20^;
Available nitrogen (kg ha^−1^)	320.5	Alkaline permanganate method^21^
Available phosphorus (kg ha^−1^)	19.7	Extraction with 0.5 M NaHCO3^22^
Available potassium (kg ha^−1^)	170.2	Flame photometer^19^

## Weather conditions

In the entire crop-growing period of 2019 and 2020, the weather was found variable. Data was obtained from the Agromet Field Unit, SKUAST-K, Srinagar as mean data. Precipitation of 371.5 mm and 303.0 mm, the minimum temperature from 9.571 to 18.290 ^◦^C and 6.711 to 18.962 ^◦^C, maximum temperature from 23.363 to 31.931 ^◦^C and 23.140 to 33.941 ^◦^C were recorded during the 2019 and 2020 crop growth duration. Also, values of mean relative humidity (minimum) from 38.570 to 70.431% and 36.570 to 75.293% and the average relative humidity (maximum) from 64.431 to 91.000% and 59.711 to 87.713% were recorded in 2019 and 2020, respectively.

## LCC-based nitrogen application

Leaf Color Chart (LCC) was developed by IRRI in collegiality with the Philippine Rice Research Institute (Manila). The technique of LCC is easy to handle, non-destructive, favorable for decisiveness and necessary for the requirements of nitrogen in crops. LCC used six color strips (from yellowish green to dark green) contriving with veins that match the maize crop leaves. It was used for the management of nitrogen, including five green strips that ranged from yellow-green to dark green, with each label marked from 1 to 5. Readings of LCC were taken every 4 days from the top expanded leaves of 10 healthy plants randomly. The middle portion of the leaf was matched with the leaf color chart to obtain the value and due care was taken while matching the color of the leaf with the chart by blocking the sunlight. When the LCC values fall below the critical limit in 5 leaves out of 10, nitrogen as per the treatments was applied. The final split was given at the tasselling stage (Table 2).

**Table 2:** Quantification of nitrogen application (kg ha^–1^) for the year 2019 and 2020 in maize field.

Treatments	SMH-2	Kanchan-45	Vivek-45	Number of splits
T_1_	-	-	-	-
T_2_	150	150.0	150.0	3
T_3_	80.0	80.0	80.0	4
T_4_	90.0	90.0	90.0	3
T_5_	100.0	100.0	100.0	5
T_6_	120.0	120.0	120.0	4
T_7_	120.0	120.0	120.0	6
T_8_	150.0	150.0	150.0	5

(where SMH-2 is Shalimar Maize Hybrid-2).

## Experimental design and treatment details

Three maize varieties (Shalimar Maize Hybrid-2 (SMH-2), Kanchan-517 and Vivek-45) with eight nitrogen application rates (T_1_: Control, T_2_: Recommended Nitrogen (120 kg N ha^–1^), T_3_: 25% N as basal LCC @20 (≤ 3) kg N ha^−1^, T_4_: 25% N as basal LCC @30 (≤ 3) kg N ha^−1^, T_5_: 25% N as basal LCC @20 (≤ 4) kg N ha^−1^, T_6_: 25% N as basal LCC @30 (≤ 4) kg N ha^−1^, T_7_: 25% N as basal LCC @20 (≤ 5) kg N ha^−1^ and T_8_: 25% N as basal LCC @30 (≤ 5) kg N ha^−1^ were evaluated in a split plot design (SPD) that was replicated thrice. The field was laid into main and subplot treatments. Eight subplots were formed out of each main plot treatment for eight different nitrogen management practices. Field preparation included two ploughings with a desirable length followed upon by leveling with a planker. Then after, 1st replication borders were made, then main plot borders, followed by subplot borders. A spacing of 75 cm × 20 cm was maintained during sowing. For nutrient management of the crop, N was given to the crop according to the treatments by employing a leaf color chart (LCC); half N, whole P and K were administered during sowing, whereas half of the nitrogen was given in equal proportions during the knee-high and tasseling stages. The crop was given nitrogen through urea, phosphorus through diammonium phosphate and potassium through muriate of potash. For Turcicum leaf blight, seed was treated with Captan + Bavistin in a 1:1 ratio @ 2 kg^−1^ and for brown strip downy mildew, seed was treated with Metalaxyl 35 SD @4 g Kg^−1^. For proper weed management of the crop, atrazine herbicide (0.75 kg a.i. ha^−1^) was sprayed at 3DAS followed by one manual hand weeding at 21DAS.

## Biometric crop observations

LAI was recorded from the formula as given in Eq. 1:

1 $$\:\text{L}\text{A}\text{I}=\frac{Tla}{Ga}$$

where, Tla is total leaf area in cm^2^ and Ga is Ground area in cm^2^.

Readings were always taken from the net plot treatment. The crop was harvested with the help of a sickle followed by shelling with the help of a sheller. For recording grain yield, at the time of harvesting, cobs were separated from husks and were sun-dried to about 15 per cent moisture content, and then after grains were removed by shelling, grain yield was computed in kg ha^–1^ and then converted to quintals per hectare. The harvest index was calculated from the formula as given in Eq. 2:

2 $$\:\text{H}\text{I}\:=\frac{\text{G}\text{Y}}{\text{B}\text{Y}}x\:100$$

where, HI is Harvest index, GY is Grain yield and BY is Biological yield.

### Computation of various NUE

Agronomic efficiency of Nitrogen (AE in kg grain kg^–1^N).

AE was computed as per Cassman et al.^23^ (Eq. 3).

3 $$\:\text{A}\text{E}\:=\frac{\left(\text{G}\text{Y}1\right)\:-\:\left(\text{G}\text{Y}2\right)}{\text{Q}\text{N}}$$

where, GY_1_ is yield in N-fertilized plots; GY_2_ is yield in zero-N plot; QN is Quantity of N fertilizer applied in N-fertilized plot.

Physiological efficiency of Nitrogen (PE in kg grain kg^–1^ N uptake).

PE was computed as per Baligar et al.^24^ (Eq. 4).

4 $$\:\text{P}\text{E}\:=\frac{\left(\text{G}\text{Y}1\right)\:-\:\left(\text{G}\text{Y}2\right)}{\left(\text{T}\text{N}1\right)-\left(\:\text{T}\text{N}2\right)}$$

where, GY_1_ is yield in N-fertilized plots; GY_2_ is yield in zero-N plot; TN_1_ is Total N uptake in N fertilized plot; TN_2_ is Total N uptake in Zero-N plot.

Apparent nutrient recovery of Nitrogen (REN in %).

REN was computed as per Cassman et al.^23^ (Eq. 5).

5 $$\:\text{R}\text{E}\text{N}\:=\frac{\left(\text{T}\text{N}1\right)-\left(\:\text{T}\text{N}2\right)}{\text{Q}\text{N}}$$

where, TN_1_ is Total N uptake in N fertilized plot; TN_2_ is Total N uptake in Zero-N plot; QN is Quantity of N fertilizer applied in N-fertilized plot.

Partial factor productivity (PFPN in kg grain kg^−1^ N) of Nitrogen.

PFPN was computed as per Cassman et al.^23^ (Eq. 6).

6 $$\:\text{P}\text{F}\text{P}\text{N}\:=\frac{\text{G}\text{Y}}{\text{G}\text{N}}x\:100$$

Where, GY is yield in N fertilized plot, QN is Quantity of fertilizer N applied.

### Statistical analysis

Standard statistical procedures were applied to the split-plot design for computing ANOVA in the current experiment for analyzing the impact of maize hybrids and nitrogen applied. This was done by employing R software version 4.2.0 (R Core Team, 2013)^25^. The statistical model of this field experiment was:

$$Yijk=\mu+\alpha\:i+\beta\:j+(\alpha\:\beta)ij+\epsilon\:ijk$$

where

*µ* is the overall mean; *αi*is the effect of the i^th^ level of the main plot factor; *βj* is the effect of the j^th^ level of the subplot factor; (*αβ*)*ij* is the interaction effect between the i^th^ level of the main plot factor and the j^th^ level of the subplot factor and $\epsilon$*ijk* is the random error term associated with the k^th^ observation within the (i, j)-^th^ combination.

## Results

### Growth parameters

The pooled data indicated that SMH-2 gave maximum plant height (189.3 cm) during the harvest stage compared to the varieties Vivek-45 and Kanchan-517 (Table 3). Among LCC values, T_8_ showed the highest plant height of 182.10 cm. The lowest plant height (155.1 cm) at harvest was observed with T_1_. Data on periodic leaf area index indicated that LAI differed significantly among three maize hybrids, and it manifested an increasing trend up to 75 DAS and then showed a declining trend till the harvest (Table 3). SMH-2 recorded significantly higher LAI of 4.9 cm compared to Kanchan-517 and Vivek-45. Besides, T_8_ and T_7_ showed significantly maximum periodic leaf area index (LAI) values among all other treatments.

**Table 3:** Plant height and periodic leaf area index (LAI) in different maize hybrid varieties as determined by precision management of nitrogen through LCC (pooled over 2019 and 2020).

Treatments	Plant height (cm)	Periodic Leaf area index (DAS)					
	Harvest	30	45	60	75	90	105
Hybrids							
SMH-2	189.4	1.5	2.5	4.4	4.9	3.6	2.4
Vivek-45	171.1	1.4	2.3	4.2	4.7	3.3	2.0
Kanchan-517	155.8	1.3	2.1	4.1	4.5	3.1	1.7
LSD (5%)	8.1	0.02	0.08	0.14	0.17	0.12	0.14
Nitrogen management							
T_1_	155.1	0.9	1.2	3.2	3.7	2.2	1.0
T_2_	171.2	1.4	2.2	4.3	4.7	3.3	2.1
T_3_	170.1	1.4	2.2	4.1	4.6	3.2	1.9
T_4_	172.3	1.4	2.3	4.2	4.7	3.3	2.1
T_5_	173.5	1.5	2.4	4.3	4.9	3.5	2.3
T_6_	175.1	1.5	2.5	4.4	5.0	3.5	2.3
T_7_	177.1	1.6	2.6	4.5	5.1	3.6	2.4
T_8_	182.1	1.6	2.8	4.7	5.2	3.8	2.6
LSD (5%)	2.5	0.04	0.05	0.10	0.15	0.14	0.12

(SMH-2 is Shalimar Maize Hybrid-2).

### Yield attributes

Pooled data manifested that maize varieties significantly influenced yield attributes (Table 4). Data disseminated that the number of rows cob^−1^ (13.6), the number of grains row^−1^ (26.6), the number of grains cob^–1^ (348.9), cob length (16.2 cm), and cob girth (10.25 cm) were recorded as significantly highest in SMH-2 in contrast to Kanchan-517 and Vivek-45, respectively, with the number of cobs plant^–1^ and seed index that were not significantly influenced by different maize hybrids. However, the higher numerical values for the number of cobs plant^–1^ and seed index (1.3 and 25.5 g) were recorded in SMH-2 in contrast to Kanchan-517 and Vivek-45.

Different LCC values recorded statistically significant impacts on different yield attributes (Table 4). Maximum the number of cobs plant^–1^ (1.4), the number of rows cob^–1^ (14.3), the number of grains row^–1^ (28.1), the number of grains cob^–1^ (383.9), cob length (18.4 cm), cob girth (11.6 cm), seed index (26.5 g) were recorded in T_8_ as compared to other nitrogen treatments. The lowest values of different yield attributes were observed in the control treatment (T_1_).

### Grain and straw yield

**Fig. 1:**
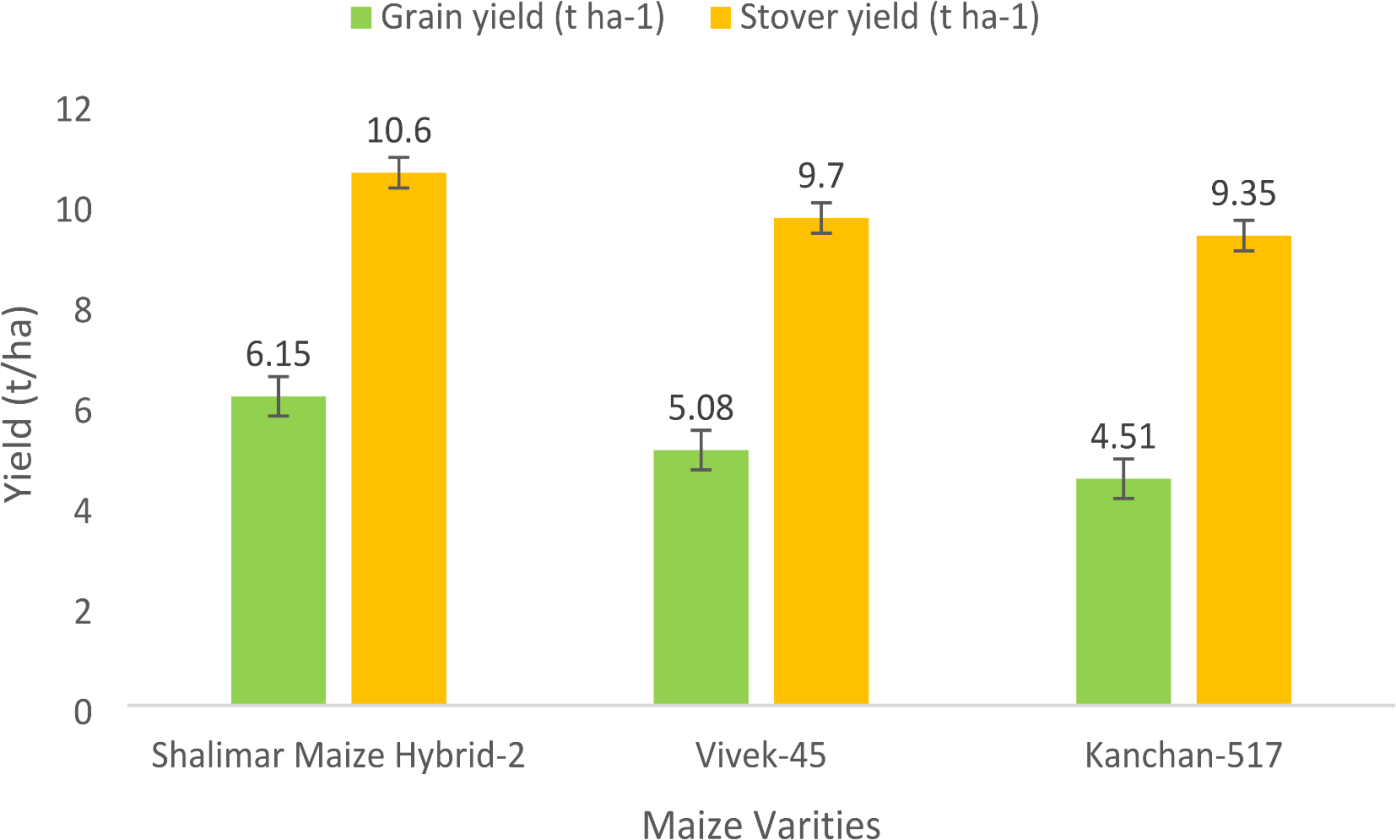
Grain and stover yield as determined by maize varieties (Pooled over 2019 and 2020). (Error bars indicate standard error).

**Fig. 2:**
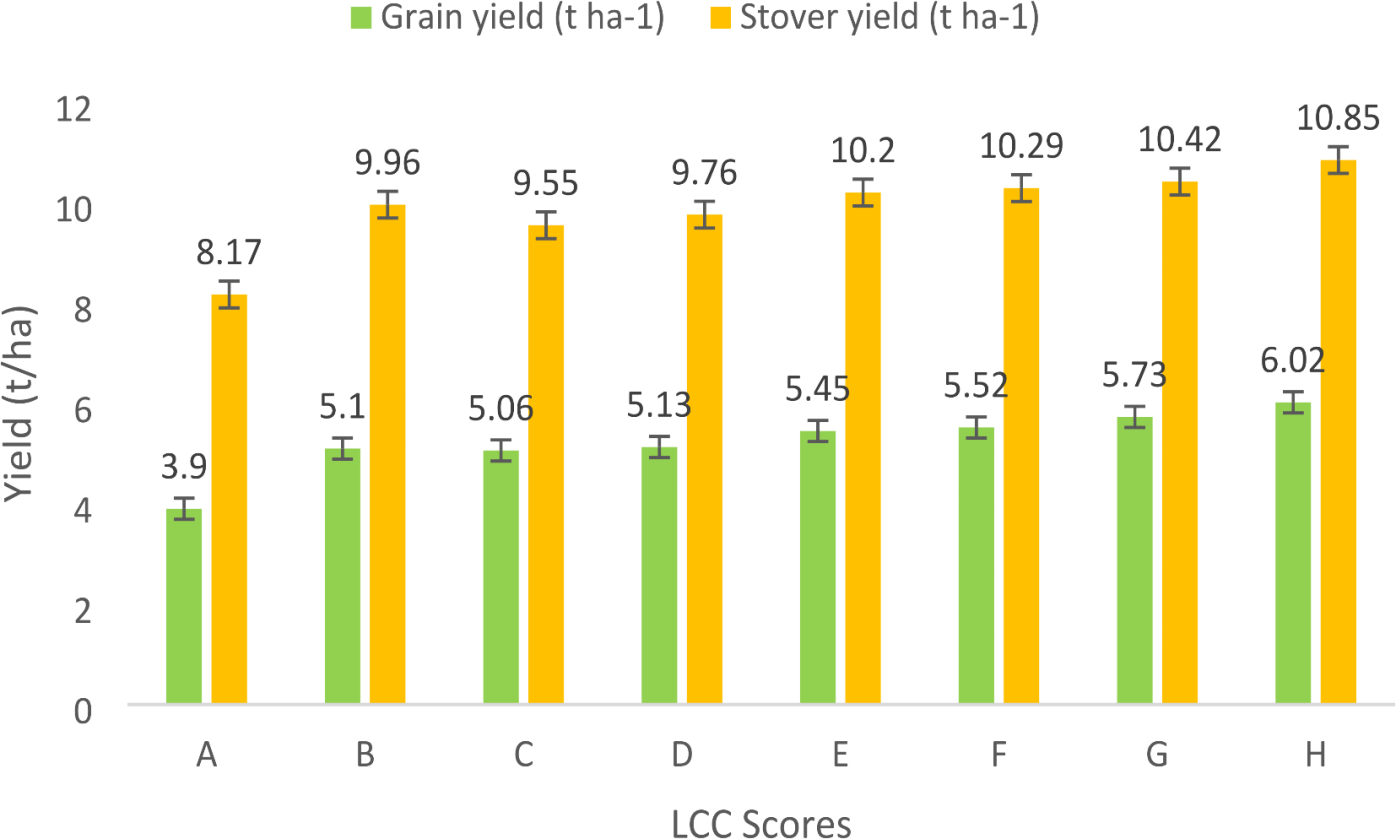
Agronomic Efficiency (AE) in kg grain kg^−1^ N/ Apparent Nutrient Recovery (REN)in %, Partial Factor Productivity (PFPN) in kg grain kg^−1^ N / Physiological Efficiency (PE) in kg grain kg^−1^ N uptake of different maize hybrid varieties pooled over 2019 and 2020 (where SMH-2 -Shalimar Maize Hybrid-2).

**Fig. 3:**
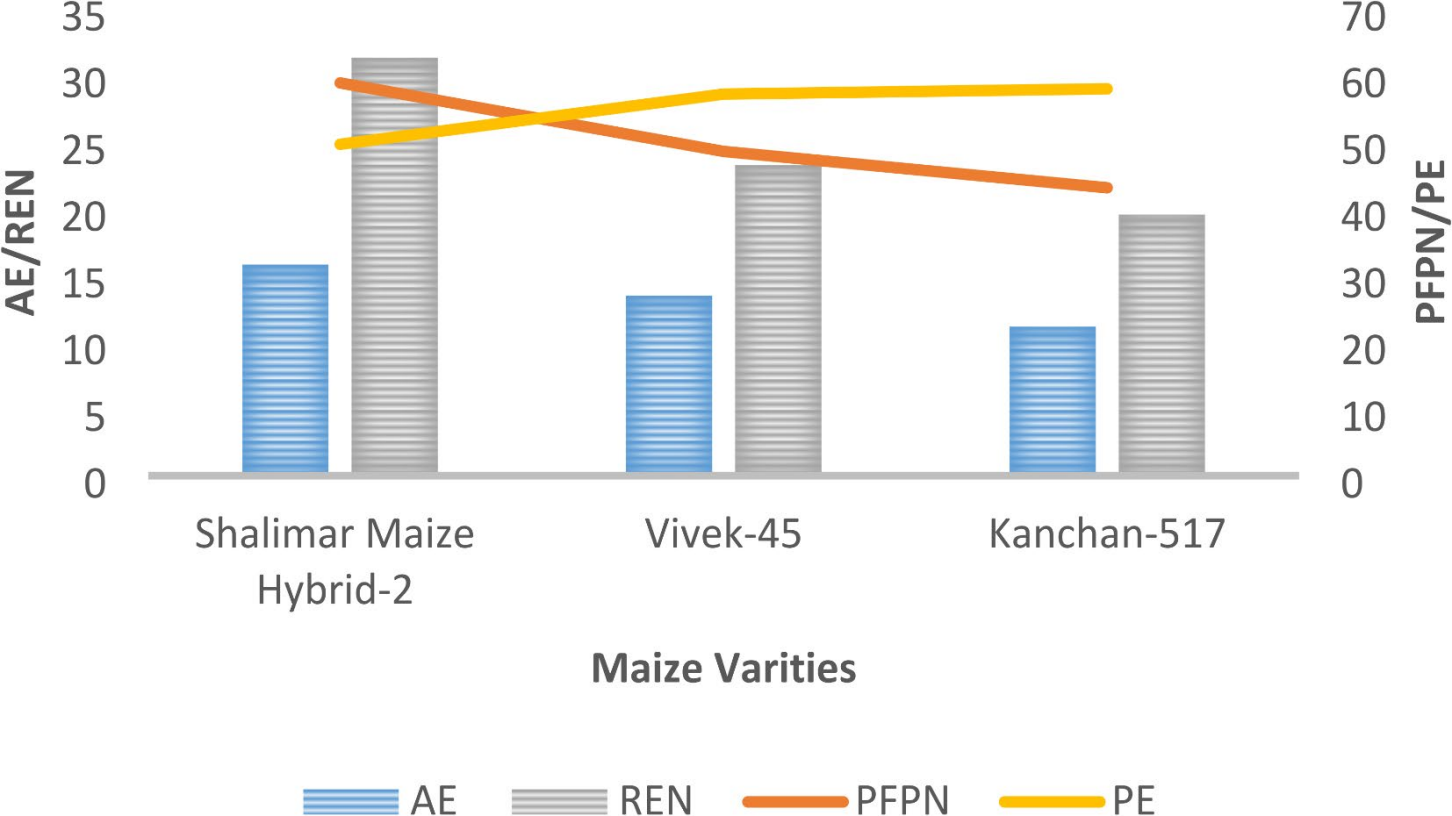
Agronomic Efficiency (AE) in kg grain kg^−1^ N/ Apparent Nutrient Recovery (REN)in %, Partial Factor Productivity (PFPN) in kg grain kg^−1^ N / Physiological Efficiency (PE) in kg grain kg^−1^ N uptake of different LCC scores pooled over 2019 and 2020 (where A- T_2_; B- T_3_; C- T_4_; D- T_5_; E- T_6_; F- T_7_; G- T_8_).

**Fig. 4:**
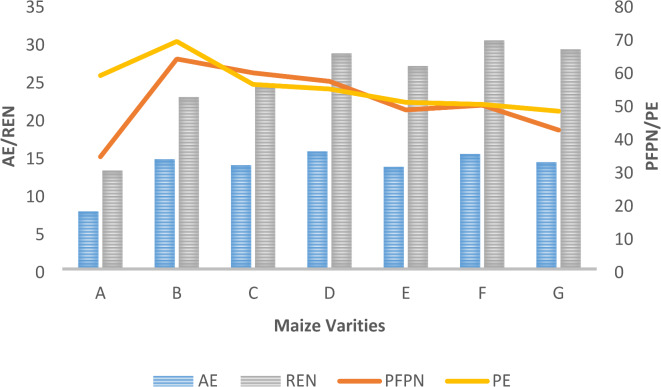
Grain and stover yield as determined by LCC scores (Pooled over 2019 and 2020). (where, A- T 1 ; B- T 2 ; C- T 3 ; D- T 4 ; E- T 5 ; F- T 6 ; G- T 7 ; H- T 8 ).

Maize hybrid varieties were statistically significantly influenced by grain yield and stover yield (Fig. 1). The pooled data indicated that SMH-2 recorded a maximum grain yield of 6.1 t ha^–1^ in comparison to Kanchan-517 (4.5 t ha^–1^) and Vivek-45 (5.1 t ha^–1^) in both consecutive years. Grain yield showed remarkable variation at various LCC scores (Figs. 2 and 3). Among different LCC levels, T_8_ recorded a significantly higher grain yield of 60.20 q ha^–1^ (Fig. 4), whereas the lowest value was observed in T_1_ (3.9 t ha^–1^). The straw yield recorded in Shalimar Maize Hybrid-2 was 10.6 t ha^–1^, and it was 9.7 and 9.3 t ha^–1^ in Vivek-45 and Kanchan-517, respectively. Straw yield was significantly influenced by LCC-based nitrogen application (Fig. 4). Treatment T_8_ recorded the highest straw yield (108.50 q ha^–1^) and the least straw yield was found under T_1_ (81.76 q ha^–1^), respectively.

The harvest index was statistically significantly affected by maize hybrids over the years (Table 4). SMH-2 was found to have the highest harvest index of 36.5%, whereas Vivek-45 and Kanchan-517 recorded harvest indees of 34.2 and 32.4%, respectively. LCC-based N applications were found to have higher harvest index values in comparison to recommended nitrogen. T_8_ showed the harvest index value of 35.5%; however, the least harvest index of 32.2% was recorded in T_1_.

**Table 4:** Yield attributes and Harvest Index in different maize hybrid varieties as determined by precision management of nitrogen through LCC (pooled over 2019 and 2020).

Treatments	Cobs plant^−1^	Rows cob^−1^	Grains row^−1^	Grains Cob^−1^	Cob length(cm)	Cob Girth (cm)	Sd. Ix. (g)	Harvest index
Hybrids								
SMH-2	1.4	13.6	26.6	349.0	16.2	10.3	25.6	36.5
Vivek-45	1.3	12.4	23.8	271.4	14.8	9.0	25.4	34.3
Kanchan-517	1.3	10.4	21.4	217.0	13.7	8.0	25.3	32.5
LSD (5%)	NS	0.4	1.0	14.0	0.9	0.8	NS	1.3
Nitrogen management								
T_1_	1.2	10.6	18.9	189.7	12.1	6.2	24.1	32.3
T_2_	1.2	11.4	22.8	248.3	13.5	8.1	24.9	33.7
T_3_	1.3	10.9	22.4	233.2	13.3	8.1	25.0	34.5
T_4_	1.3	11.6	23.2	255.6	13.9	8.7	25.1	34.3
T_5_	1.3	12.0	24.6	280.8	15.2	9.4	25.6	34.7
T_6_	1.3	12.7	25.3	305.4	15.9	10.0	25.9	34.8
T_7_	1.4	13.4	26.3	336.1	16.9	10.6	26.3	35.3
T_8_	1.4	14.4	28.1	384.0	18.5	11.6	26.6	35.6
LSD (5%)	NS	0.5	1.1	17.9	0.6	0.5	0.7	1.4

(where SMH-2 - Shalimar Maize Hybrid-2).

### N use efficiency

#### Agronomic efficiency of nitrogen (AE kg grain/kg N applied)

Different maize hybrids (Fig. 2) recorded a non-significant effect while LCC scores (Fig. 3) reported a significant impact on AE over the years. The highest agronomic efficiency of 15.4 kg grain/kg N was recorded in T_5,_ which was at par with T_7_, and the lowest values of 7.6 kg grain/kg N were recorded in T_2,_ respectively.

### Physiological efficiency of nitrogen (PE kg grain/kg N uptake)

Different LCC treatments (Fig. 3) reported significant effects, whereas different maize hybrids (Fig. 2) reported non-significant effects on physiological efficiency. Physiological efficiency was highest in T_3_ (68.5 kg grain/kg N uptake), whereas, T_8_ recorded a physiological efficiency of 47.5 kg grain/kg N uptake, respectively.

### Apparent nutrient recovery of nitrogen (REN %)

Different maize hybrids showed significant impact on apparent nutrient recovery over the years of experimentation (Fig. 2). SMH-2 reported an apparent nutrient recovery of 31.3%, which was the highest as compared to Kanchan-517 and Vivek-45, respectively. Also, T_7_ at par with T_8_ reported a higher apparent nutrient recovery of 30.1%, whereas T_2_ reported the lowest apparent nutrient recovery of 12.9% (Fig. 3).

### Partial factor productivity of Nitrogen (PFPN kg grain kg-1 N applied)

Different maize hybrids (Fig. 2) and LCC scores (Fig. 3) reported a significant effect on partial factor productivity. SMH-2 recorded the highest partial factor productivity (58.8 kg grain/kg N) as compared to other maize hybrids over the years of experimentation. Nitrogen management through T_3_ reported the highest values of partial factor productivity (63.2 kg grain/kg N), whereas the lowest was reported in T_2_ (33.8 kg grain/kg N), respectively.

### Soil nutrient status

Soil pH, EC and organic Carbon didn’t record any significant effect of the experiment. Maize hybrids and different LCC scores were having a non-significant impact on pH, EC and OC values recorded.

### Available nitrogen (kg ha–1)

Pooled data of two years on available nitrogen (Table 5) revealed that in plots where Shalimar Maize Hybrid-2 was planted, the soil available nitrogen was 241.4 kg ha^–1^ in comparison to plots of Kanchan-517 and Vivek-45, where the available soil nitrogen was 257.0 kg ha^–1^ and 268.7 kg ha^–1^, respectively, specifying that SMH-2 was extracting more nitrogen from the soil in comparison to other hybrids. Available nitrogen status in soil for T_8_ was 275.0 kg ha^–1^, whereas the available nitrogen in soil in T_7_ was 269.0 kg N ha^–1^, respectively.

**Table 5:** Available nitrogen (kg ha^−1^), available phosphorus (kg ha^−1^) and available potassium (kg ha^−1^) in different maize hybrid varieties as determined by precision management of nitrogen through LCC (pooled over 2019 and 2020).

Treatments	Available nitrogen	Available phosphorus	Available potassium
Hybrids			
SMH-2	241.4	21.9	152.3
Vivek-45	257.1	19.6	159.8
Kanchan-517	268.7	22.5	166.9
LSD (5%)	7.4	0.7	4.9
Nitrogen management			
T_1_	227.5	23.8	158.8
T_2_	271.8	22.3	166.8
T_3_	239.4	23.4	171.4
T_4_	246.9	21.5	164.1
T_5_	256.3	21.3	158.8
T_6_	260.1	20.5	153.8
T_7_	269.1	20.3	152.8
T_8_	275.1	18.1	151.3
LSD (5%)	9.2	1.0	8.2

(where SMH-2 is Shalimar Maize Hybrid-2).

### Available phosphorus (kg ha-1)

The description of the data (Table 5) showed that in plots where ShMH-2 was planted, the soil available phosphorus was 21.9 kg ha^–1^ in comparison to plots of Kanchan-517 and Vivek-45, where the available soil phosphorus recorded was 19.6 kg ha^–1^ and 22.5 kg ha^–1^, respectively, signifying that SMH-2 extracted more P from the soil in comparison to other varieties. Treatment T_8_ reported available P of 18.1 kg ha^–1^, whereas T_7_ recorded available phosphorus in soil as 20.3 kg ha^–1^.

### Available potassium (kg ha-1)

Description of data (Table 5) unveiled that plots where SMH-2 was planted, the soil available phosphorus was 152.3 kg ha^–1^ as compared to plots of Kanchan-517 and Vivek-45 where the available soil potassium recorded were 159.8 kg ha^–1^ and 166.9 kg ha^–1^, respectively. Further, T_8_ reported to have 151.3 kg ha^–1^ available K.

#### Economics

The economic analysis of the pooled data showed that treatment T_8_ (25% N as basal LCC @30 (≤ 5) kg N ha^–1^) reported to have the highest gross returns (***$***1548.9), net returns (***$***1001) and benefit-cost ratio (1.83) followed by T_7_ (25% N as basal LCC @20 (≤ 5) kg N ha^−1^) (Table 6). The quantity of nitrogen applied in T_8_ (25% N as basal LCC @30 (≤ 5) kg N ha^−1^) was the same as that of the recommeneded dose but with two additional splits, whereas the amount of N applied in T_7_ (25% N as basal LCC @20 (≤ 5) kg N ha^−1^) was 30 kg ha^−1^ less (saving) than that of the recommeneded dose with 3 additional splits than the recommended dose. SMH-2 recorded the highest gross returns (***$***1577.3), net returns (***$***1037.2) and B: C ratio (1.92) as compared to other hybrids, respectively. The relative economics of leaf color chart (LCC)-based nitrogen management in maize prove to be promising, as this cost-effective visual tool not only aids in optimizing nitrogen applications, potentially reducing fertilizer costs, but also contributes to sustainable farming practices by minimizing environmental impacts associated with over-fertilization, making it a financially prudent and environmentally responsible approach for maize cultivation.

**Table 6:** Economic in different maize hybrid varieties as determined by precision management of nitrogen through LCC (pooled over 2019 and 2020).

Treatments	CC ($ ha^–1^)	GR ($ ha^–1^)	NR($ ha^–1^)	B: C ratio
Hybrids				
SMH-2	540.8	1577.3	1037.2	1.92
Vivek-45	540.8	1313.5	773.4	1.43
Kanchan-517	540.8	1176.4	636.3	1.18
Nitrogen mangement				
T_1_	527.8	1030.5	502.8	0.95
T_2_	548.6	1321.8	778.6	1.42
T_3_	537.0	1307.7	770.6	1.44
T_4_	538.6	1328.1	789.6	1.47
T_5_	540.1	1407.9	867.8	1.61
T_6_	543.2	1425.4	882.2	1.62
T_7_	543.2	1475.5	932.4	1.72
T_8_	547.8	1548.9	1001.1	1.83

**A**_**1**_ = Shalimar maize Hybrid-2; **A**_**2**_ = Vivek-45; **A**_**3**_ = Kanchan-517; T_1_: Control, T_2_: Recommended Nitrogen, T_3_: 25% N as basal LCC @20 (≤ 3) kg N ha^−1^, T_4_: 25% N as basal LCC @30 (≤ 3) kg N ha^−1^, T_5_: 25% N as basal LCC @20 (≤ 4) kg N ha^−1^, T_6_: 25% N as basal LCC @30 (≤ 4) kg N ha^−1^, T_7_: 25% N as basal LCC @20 (≤ 5) kg N ha^−1^ and T_8_: 25% N as basal LCC @30 (≤ 5) kg N ha^−1^ ; CC is cost of cultivation; GR is gross returns, NR is net returns).

## Discussion

Nitrogen is a sine qua non for the production of crops, being indispensable in keeping up crop growth and yield. Non-synchronous application of N with the crop needs leads to lower N use efficacy^26^. Blanket applications of fertilizers seem to be quotidian in Asian fields that lead the way for the non-competence of management of N^27^. Fertilization of maize crop being much the same^28^also has lower N use efficiency vis-à-vis surfeit application of N, disorganized splitting of N. Synchronous application of N with the crop growth is one of the restorative measures in this case^29^. Conjugation of supply of N with its demand in the crop is the blueprint for improving N use proficiency (28). It can be only carried out by successive precision management of N upon timely gauging of N status within the plant and when the N falls below the critical levels^30^. Tools like chlorophyll meters and LCC are equally helpful in determining the chlorophyll content of leaves that give us the measure of N content of leaves as and when used appropriately. Nitrogen management with the correct splitting schedule in conjunction with the SPAD meter and LCC is another effective approach for elevated N use efficiency and thus defending the research done. The implications of our study extend beyond mere nitrogen optimization, as the differential responses of maize cultivars to LCC-based nitrogen management illuminate the intricate interplay between genetic factors, environmental conditions, and nutrient dynamics, offering valuable insights for breeders and agronomists alike.

Upon careful scrutiny of the results, it was found that SMH-2 set down higher growth and yield parameters and eventually higher yield in comparison to Kanchan-517 and Vivek-45 owing to the varietal differences in their genetic makeup. Supplementing N to the crop in adequate levels amplifies cell division and cell elongation, leading the way to elongation of internodes and ultimately plant height and other growth parameters. Higher uptake and availability of N in T_8_ (25% N as basal LCC @30 (≤ 5) kg N ha^−1^) and T_7_ (25% N as basal LCC @20 (≤ 5) kg N ha^−1^) that acts as a substrate for the manufacturing of those organic compounds, which are the components of chlorophyll within the plant, might have contributed to their higher growth, yield parameters and yield in comparison to the rest of the treatments^31,32^. Moreover, synchronous application of N to maize as and when required with correct splitting wodge vis-à-vis higher dosage of N owned to higher yield and yield attributes of maize^33^. Disparity in the growth components of different varieties corresponded to disparities in the yield attributes. Similar results were also endowed by Tarafdar et al.^34^. Also, higher dry matter and higher cobs per plant and higher grains per row were statistically higher in SMH-2 as compared to other two varieties that became the reason for higher yield in SMH-2. Similar findings were delineated by Jyothsna et al.^35^.

Treatment T_8_ (25% N as basal LCC @30 (≤ 5) kg N ha^–1^) and T_7_ (25% N as basal LCC @20 (≤ 5) kg N ha^–1^) were found to have statistically more grain yield in comparison to other LCC scores, which probably was due to an increase in the number of nitrogen splits. The results validate with the findings of Jyothsna et al.^35^. , Bhat et al.^36^ and Bhat et al.^37^. With an increase in the number of splits, losses of nitrogen through volatilization and denitrification decreased as nitrogen was correlated with the demand that ensued in higher grain yield. Also, the access of nutrients to the crop at each phase favored the photosynthates accumulation towards grains which also gave rise to higher grain yield and eventually higher values in number of cobs per plant and more grains per row^38^. More stover yield in SMH-2 in contrast to Kanchan-517 and Vivek-45 was registered and this was also recorded by Jyothsna et al.^35^. Among different treatments, T_8_ and T_7_ recorded more stover yield in contrast to other LCC scores. Stover yield of T_5_ (25% N as basal LCC @20 (≤ 4) kg N ha^−1^) and T_6_ (25% N as basal LCC @30 (≤ 4) kg N ha^−1^) at both levels was endowed to be at par with T_2_ (recommended dose) but was maximum in comparison to both. More stover yield in LCC 5 in comparison to other scores might have been because of increased vegetative growth in LCC 5 score. Dry matter accumulation was found to be correlated to the dosage of N at knee high stage and eventually resulted in a greater number of cobs per plant. The results corroborate the discoveries of Mathukia et al.^38^. The harvest index signifies the accumulation of photosynthates to sink via the source. Shalimar Maize Hybrid-2 noted more higher harvest index as compared to the other two varieties in consecutive years. Among different LCC scores, T_8_ and T_7_ recorded more HI in comparison to other LCC scores. These results were found in close proximity with Mathukia et al.^38^.

The effect of maize hybrids on apparent nitrogen recovery and partial factor productivity was found to be significant in comparison to physiological efficiency and agronomic efficiency. Partial factor productivity (PFPN) was found to be high in SMH-2 in contrast to other varieties. The reason might be higher yield, N accumulation and uptake of NH4^+^. Results were in line with Banerjee et al.^39^. Among different LCC scores, highest partial factor productivity and physiological efficiency was recorded by T_3_ (25% N as basal LCC @20 (≤ 3) kg N ha^−1^) and highest apparent recovery of nitrogen in T_8_ and T_7_ and maximum agronomic efficiency in T_5_ and T_7_ whereas lowest values for apparent N recovery (REN) and agronomic efficiency (AE) were recorded by T_2_. NUE was found to be indirectly correlated with LCC values. Thus, with each increment in LCC values, there was decrease in NUE. LCC 3 recorded more NUE in contrast to other LCC values. Applying nitrogen during the peak nutritive demand for N will result in more NUE and eventually less losses in nitrogen. This decrease in NUE with an increase in N applied might have been because of higher levels of other nutrients beyond certain levels as stated by the law of diminishing returns. Parallel results have been endowed by Banerjee et al.^39^ and Subedi et al.^40^. Cereal crops like maize generally show a higher response of N with a lower dosage of N and thus record a linear response and then a quadratic response for lower inputs of fertilizer. Thus, it was highlighted that an increase in the amount of application of N decreased NUE.

In economics calculated for both years, Shalimar Maize Hybrid-2 recorded a higher benefit-cost ratio, which could be due to a higher yield^41^. Synchronization of nitrogen application with its demand and higher yields in LCC 5 might be the reason for the higher net returns. Bhuiya et al.^42^, Bhat et al.^43^. drew parallel results.

While LCC has demonstrated its efficacy in real-time nitrogen management, our discussion prompts consideration of its broader role in precision agriculture, urging researchers and practitioners to explore the integration of this visual tool with advanced technologies for a comprehensive and scalable approach to cultivar-specific nutrient optimization. The nuanced response of maize cultivars to LCC-based nitrogen management introduces an exciting avenue for targeted agronomic interventions, paving the way for the development of precision strategies that not only enhance yield but also minimize environmental impacts through judicious nutrient application.

## Conclusions

Shalimar Maize Hybrid-2 with N application as T_8_ (25% N as basal LCC @30 (≤ 5) kg N ha^−1^) and T_7_ (25% N as basal LCC @20 (≤ 5) kg N ha^−1^) was economically acceptable and ought to be promulgated under temperate ecology. Further, there was a saving of 30 kg N ha^−1^ and a higher yield with T_7_ (25% N as basal LCC @20 (≤ 5) kg N ha^−1^) as compared to the recommended dose of nitrogen. Therefore, the leaf color chart came to light as being an easy, portable and affordable tool for efficient management of Nitrogen in maize under the temperate ecology of Kashmir. Besides, LCC-based nitrogen management in these maize cultivars reveals a promising avenue for precision agriculture. The cultivar-specific responses observed underscore the need for tailored nitrogen strategies, advocating for the integration of LCC as a practical and cost-effective tool in optimizing nutrient applications.

## Data Availability

The datasets used and/or analyzed during the current study are available from the corresponding author on reasonable request.
